# Mast Cells in Stress, Pain, Blood-Brain Barrier, Neuroinflammation and Alzheimer’s Disease

**DOI:** 10.3389/fncel.2019.00054

**Published:** 2019-02-19

**Authors:** Duraisamy Kempuraj, Shireen Mentor, Ramasamy Thangavel, Mohammad E. Ahmed, Govindhasamy Pushpavathi Selvakumar, Sudhanshu P. Raikwar, Iuliia Dubova, Smita Zaheer, Shankar S. Iyer, Asgar Zaheer

**Affiliations:** ^1^Harry S. Truman Memorial Veterans’ Hospital (VA), U.S. Department of Veterans Affairs, Columbia, MO, United States; ^2^Department of Neurology and the Center for Translational Neuroscience, School of Medicine, University of Missouri, Columbia, MO, United States

**Keywords:** Alzheimer’s disease, amyloid plaques, chronic stress, corticotropin releasing hormone, mast cells, neurodegenerative disease, neuroinflammation

## Abstract

Mast cell activation plays an important role in stress-mediated disease pathogenesis. Chronic stress cause or exacerbate aging and age-dependent neurodegenerative diseases. The severity of inflammatory diseases is worsened by the stress. Mast cell activation-dependent inflammatory mediators augment stress associated pain and neuroinflammation. Stress is the second most common trigger of headache due to mast cell activation. Alzheimer’s disease (AD) is a progressive irreversible neurodegenerative disease that affects more women than men and woman’s increased susceptibility to chronic stress could increase the risk for AD. Modern life-related stress, social stress, isolation stress, restraint stress, early life stress are associated with an increased level of neurotoxic beta amyloid (Aβ) peptide. Stress increases cognitive dysfunction, generates amyloid precursor protein (APP), hyperphosphorylated tau, neurofibrillary tangles (NFTs), and amyloid plaques (APs) in the brain. Stress-induced Aβ persists for years and generates APs even several years after the stress exposure. Stress activates hypothalamic-pituitary adrenal (HPA) axis and releases corticotropin-releasing hormone (CRH) from hypothalamus and in peripheral system, which increases the formation of Aβ, tau hyperphosphorylation, and blood-brain barrier (BBB) disruption in the brain. Mast cells are implicated in nociception and pain. Mast cells are the source and target of CRH and other neuropeptides that mediate neuroinflammation. Microglia express receptor for CRH that mediate neurodegeneration in AD. However, the exact mechanisms of how stress-mediated mast cell activation contribute to the pathogenesis of AD remains elusive. This mini-review highlights the possible role of stress and mast cell activation in neuroinflammation, BBB, and tight junction disruption and AD pathogenesis.

## Introduction

Stress is our body’s normal physiological response to any adverse changes in our environment to deal and overcome these challenges (Bisht et al., 2018). Chronic diseases can disrupt the quality of normal life and day-to-day life activities that can lead to psychological stress. Stress induces the onset and progression of pain, cognitive disorder, and psychiatric disorders. Stress induces disease(s) and the disease (s) in turn can exacerbate the stress severity in a vicious cycle (Justice, 2018). Chronic stress due to continuous wars, military service, chronic diseases, dementia, neurotrauma, poor sleep habits, immobilization, isolation, noise, high workload, unstable job, annoying work environment, difficult spouse; acute stress due to chronic diseases, modern life conditions, immobilization, isolation, noise, physical, visual, emotional, social, environmental, temperatures (hot and cold), odors, certain foods, new challenges, competitions, presentation at work, and intermittent fasting can induce several unwanted changes in the central nervous system (CNS). These changes include cognitive disorders, neuroinflammation, altered secretion of growth factors, high proinflammatory cytokines and chemokines secretion, increased oxidative stress, blood-brain barrier (BBB) disruption, ultrastructural and molecular changes in tight junctions, neurovascular unit (NVU), gliovascular unit (GVU), changes in the brain volume, and neuroinflammation (Kempuraj et al., 2017a; Lurie, 2018). Stress can also induce the changes in the peripheral system, the CNS immune components, and affect immune cells such as mast cells.

Blood-brain barrier disruption is associated with the entry of proinflammatory cytokines, chemokines, immune and inflammatory cells in to the brain, neuroinflammation and neurodegeneration (Patel and Frey, 2015; Kempuraj et al., 2017b). Stress and immune system interact bi-directionally and enhance stress response even in the CNS (Holzer et al., 2017). Mast cell activation induces glial cells activation, neuroinflammation, stress response, and pain signals (Theoharides et al., 2012; Kempuraj et al., 2017b; Skaper, 2017; Skaper et al., 2017; Gupta and Harvima, 2018; Theoharides and Kavalioti, 2018). Stress conditions inhibit immune response, but can worsen inflammatory conditions including neuroinflammation (Esposito et al., 2001a; Karagkouni et al., 2013). In fact, most of the CNS disorders show disruption of BBB and tight junction proteins. Mast cells and neurons are closely associated both anatomically and functionally throughout the body including the CNS (Forsythe, 2019). The number, distribution and the activation status of mast cells in the brain is not constant, but varies due to environment, behavioral changes and physiological state (Forsythe, 2019). Neuroinflammation induces NVU and GVU dysfunctions in many neuroinflammatory diseases (Li et al., 2017). Increased levels of inflammatory cytokines, chemokines and microglial activation contribute to the activation of pain mechanisms (Lurie, 2018). The initial stress and pain responses protect the body, however, chronic stress and chronic pain can induce many health problems. In this mini-review, we highlight the recent knowledge on the possible role of stress, and mast cell activation in neuroinflammation, BBB and tight junction disruption, onset, progression and severity of Alzheimer’s disease (AD).

## Mast Cells, Pain, and Neuroinflammation

Mast cells are implicated in neuroprotection, pain, neuroinflammation and neurodegenerative diseases by releasing several preformed and preactivated inflammatory mediators, as well as release of newly synthesized cytokines, chemokines, and neurotoxic molecules (Gordon and Galli, 1990; Kempuraj et al., 2017b; Conti et al., 2018; Kempuraj et al., 2018a,b,c; Ocak et al., 2018; Skaper et al., 2018). IL-1 family cytokines such as IL-1β and IL-33 can activate mast cells and are implicated in inflammation including neuroinflammation but IL-37 is anti-inflammatory cytokine that can be used to treat inflammatory conditions (Tettamanti et al., 2018; Varvara et al., 2018). Neuroinflammation further leads to the release of additional inflammatory cytokines, chemokines, prostaglandins, activation of nociceptors, acute and chronic pain, headache, BBB dysfunction, neuronal excitability, and glial and neuronal damage in the CNS (Skaper et al., 2012, 2017; Skaper, 2016). Patients with neurodegenerative diseases such as AD, and Parkinson’s disease (PD), Huntington’s disease (HD) show painful symptoms, but the origin of pain is variable in these patients (de Tommaso et al., 2017; Matsuda et al., 2018).

About 38–75% of AD patients and 40–86% of PD patients also show painful symptoms in addition to other clinical disorders (de Tommaso et al., 2016). The International Association for the Study of Pain (IASP) describe, “pain is an unpleasant sensory and emotional experience associated with actual or potential tissue damage or described in terms of such damage” (de Tommaso et al., 2016). The origin of pain in neurodegenerative diseases is multifactorial involving either nociceptive or neuropathic and sometimes both. The prevalence of dementia and pain increases with aging (Defrin et al., 2015). However, severe dementia and AD dementia patients are unable to report the full extent and the severity of pain, and therefore pain symptoms are not properly treated in these patients. About 50% of community dwelling patients and about 45–83% of dementia patients living in nursing homes suffer from pain due to various causes including infections. It is not clear if the drugs such as L-dopa or riluzole used in the patients are effective in significant pain suppression (de Tommaso et al., 2017). Therefore, pain management needs careful evaluation in these neurodegenerative patients (de Tommaso et al., 2017).

**FIGURE 1:**
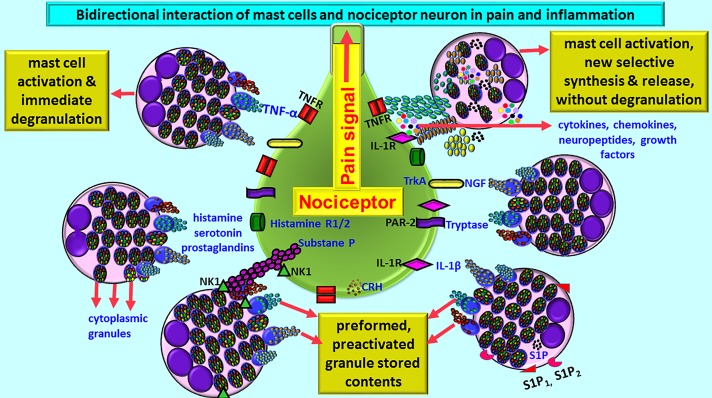
Schematic diagram shows bidirectional communications between nociceptor neuron and mast cells during pain and inflammation. Mast cells are located close to nociceptor/neurons. Several conditions activate mast cells to release preformed preactivated and granule stored neuroactive inflammatory mediators and growth factors by degranulation or release many newly synthesized neuroactive and neuroinflammatory mediators. Mast cells express several receptors including CRHR, NK1, S1P1, and S1P2 for the mediators released from the neurons and for the cytokines/chemokines and various growth factors. Similarly, nociceptor neurons also express receptors including PAR-2, TNF-R, IL-1R, histamine R1/2, and NK-1for mast cell released mediators. Histamine, serotonin, and prostaglandins released from mast cells induce pain signals. Mast cell-released inflammatory mediators and growth factors induce pain signals and inflammation in many neuroinflammatory and neurodegenerative diseases. CRHR, corticotropin-releasing hormone receptor; IL-1R, IL-1 receptor; NK-1, neurokinin-1; NGF, nerve growth factor; PAR-2, protease-activated receptor-2; S1P, sphingosine-1 phosphate; TNFR, TNF receptor; TrkA, Tropomyosin receptor kinase A.

Mast cells may either directly influence nociceptive neurons or through glial cells, based upon the location of mast cells, and pain pathways in the brain (Caraffa et al., 2018; Gupta and Harvima, 2018). Mast cell-derived TNF is known to sensitize meningeal nociceptors and induce neuroinflammation (Caraffa et al., 2018). Because of the presence of vicious positive-feedback mechanism of mast cells and glial cells activation with inflammatory mediators’ release, even a small number of mast cells can induce significant neuroinflammation in the brain. In fact, about 50% of histamine and 25% of tumor necrosis factor-alpha (TNF-α) are from the mast cells in the rat brain that can cause nociception and pain signals in the brain (Hendriksen et al., 2017; Gupta and Harvima, 2018). Mast cell activation leads to the release of many neuropeptides and inflammatory mediators including histamine, tryptase, and prostaglandins that can act on nociceptor on sensory neurons for the pain sensation (Schwartz, 1990; Levy et al., 2012; Aich et al., 2015; Kempuraj et al., 2017b). Neurons in turn release various neuropeptides, neuroinflammatory and analgesic mediators that can activate mast cells in a vicious cycle. This continuous process leads to an increased vascular permeability, chronic pain, itch, inflammation, and neuroinflammation (Gupta and Harvima, 2018; Figure 1). Mast cells are present at the nerve terminals in the periphery, meninges, and vasculature in the brain (Gupta and Harvima, 2018). Mast cells-associated histamine, tryptase, nerve growth factor (NGF), sphingosine-1 phosphate (S1P) are involved in the pain sensation. Mast cell-released histamine acts on nerve endings through histamine 1 receptor (H1R), H2R, H3R, and H4R. Therefore, anti-histaminergic drugs show significant reduction in the pain sensation in the humans. Nociceptive C and A-delta nerve fibers respond to histamine in the peripheral system and in the CNS, and transmit nociceptive signals to the thalamus and then to the cortical and subcortical areas including amygdala and striatum regions. Neuronal calcitonin gene related peptide (CGRP) induces mast cells to release histamine. Mast cell proteinase tryptase acts on nerve endings through protease-activated receptor-2 (PAR-2) and increases the release of substance P and CGRP, which in turn induce mast cell activation and release inflammatory mediators (Figure 1). Thus, increased mast cell activation is associated with high levels of tryptase associated with severe pain (Gupta and Harvima, 2018). Increased mast cell activation also increases tryptase levels in the blood. Mast cells synthesize and secrete NGF that can act again on mast cells as well as nerve endings through its receptor tropomyosin receptor kinase A (TrkA) and further release histamine and NGF from mast cells. NGF level has been shown to be increased in various inflammatory and painful conditions that are associated with increased mast cell activation. Mast cells also release S1P that can act on mast cells through S1P1 and S1P2 receptors and induce mast cell activation and degranulation (Gupta and Harvima, 2018). These mechanisms induce pain, mast cells recruitment to the site of inflammation, and chemokine release. Both NGF and S1P receptor antagonists are shown to be useful in reducing the severity of pain in inflammatory disorders. All these findings show that mast cells are involved in pain sensation including headache associated with neuroinflammation.

## Stress, Pain, and Neuroinflammation

Chronic diseases can disrupt normal life and day-to-day life activities that may lead to psychological stress. Psychological stress, diet, hormonal fluctuations, and post-traumatic stress disorder (PTSD) can induce inflammation including sterile inflammation, oxidative stress, pain, and neuroinflammation (Ferdousi and Finn, 2018; Garfin et al., 2018; Ramachandran, 2018; Rometsch-Ogioun El Sount et al., 2018). Increased BBB permeability causes edema, increased S100B expression, and neuroinflammation (Koh and Lee, 2014). Activated mast cells cause both neuronal response and vascular response, as they are close to BBB structure and neurons. Stress-induced mast cell activation in dural vasculature plays an important role in the acute and chronic headaches (Kandere-Grzybowska et al., 2003; Shelukhina et al., 2017). Psychological stress conditions activate neurons to release CGRP, substance P, and neurokinins that activate mast cells and release many inflammatory mediators. These inflammatory mediators activate nociceptors and pain mechanisms (Forsythe, 2019). Recent reports indicate that stress induces inflammation in many diseases such as allergic diseases, eczema, fibromyalgia, mast cell activation syndrome, irritable bowel syndrome, chronic fatigue syndrome, and autism, and that the intranasal administration of natural flavonoid compounds such as tetramethoxyluteolin, and luteolin with Ashwaganda could inhibit inflammation, neuroinflammation and the severity of neurodegenerative diseases (Theoharides and Kavalioti, 2018; Theoharides and Tsilioni, 2018). Further, this report also suggests that interaction of mast cells and microglia in the hypothalamus could induce stress-mediated neuroinflammation (Theoharides and Kavalioti, 2018). Other natural plant products such as thymoquinone is known to improve cognitive disorders and neuroprotective effects in cerebral small vessel disease and can protect from stress effects (Guan et al., 2018).

Chronic stress and chronic pain conditions are considered as two sides of the same coin due to the similarities between them, though they are actually different (Abdallah and Geha, 2017). Hypothalamus, hippocampus, amygdala and pre-frontal cortex (limbic system) are important in learning process. These regions process incoming nociceptive pain signals as well as the signals from stress, and make signals for the specific decision making. Limbic system and hypothalamic-pituitary adrenal (HPA) axis are interconnected and regulate stress response of the body. Both chronic stress and chronic pain affect these regions and impair the functions. Factors such as low income, poverty, uncompleted education and unsuccessful occupation account for the socioeconomic stress-mediated adverse behavior, depression, substance use, crime, and obesity (Abdallah and Geha, 2017). Both chronic stress and pain can reduce the hippocampal volume and the stress is a risk factor for developing pain in the human (Chen et al., 2010; Mutso et al., 2012; Abdallah and Geha, 2017). Dark microglia, a newly identified microglia phenotype has been associated with stress and AD (Bisht et al., 2018). These dark microglia are structurally different from ionized calcium-binding adapter molecule 1 (Iba1) expressed microglia, and they are increased at the site of microglial alteration and activation such as around amyloid plaques (APs), dystrophic neurons, triggering receptor expressed on myeloid cells 2 (TREM2), in stress, aging, and AD (Heneka et al., 2013; Zheng et al., 2017). Chronic stress can induce BBB disruption and increase neuroinflammation that can induce and worsen AD pathogenesis. BBB dysfunction causes decreased beta amyloid (Aβ) entry from brain to blood circulation. AD induces ultrastructural changes in the endothelial cells, tight junction proteins, pericytes and astrocytes, increase oxidative stress, neuroinflammation, and enhance Aβ level by increasing β and γ-secretase activities. These changes continue as positive-feedback loop and cause dementia and cognitive disorders (Cai et al., 2011, 2018). Several acute stress conditions are associated with severe headache. Migraine headaches and neuroinflammation are worsened by stress conditions (Ramachandran, 2018). Migraine is also known to induce BBB permeability (Dreier et al., 2005). Previous study has shown that acute immobilization stress induces the activation of dura mast cells in C57BL/6 mice, but not in neurokinin-1 receptor deficient (NK-1R KO) mice. Moreover, stress-induced vascular permeability was reduced in mast cell deficient mice (Kandere-Grzybowska et al., 2003). These studies show that mast cells are important in stress-mediated adverse effects in the CNS.

**FIGURE 2:**
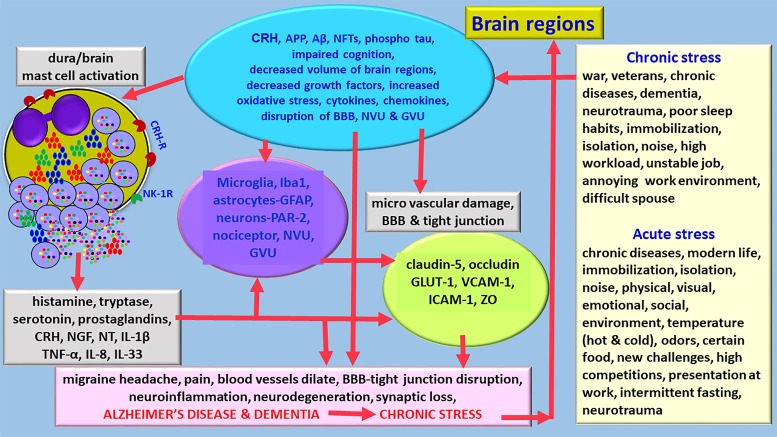
Diagram showing stress can exacerbate neuroinflammation and neurodegeneration and accelerates the pathogenesis of AD. Various chronic stress and acute stress conditions differentially activates hypothalamus and release CRH in the brain. Stress also activates immune and inflammatory response in the brain that leads to the activation of immune and inflammatory cells, and glia associated with neuroinflammatory mediator release and pain. Stress increases the generation of APP, Aβ, hyperphosphorylated tau, induces cognitive disorders, reduce brain volume, decrease growth factor expression, increases oxidative stress, and activates mast cells and neuroglia. Several mast cell-derived inflammatory mediators induce severe migraine/headache in stress conditions. Stress affects BBB functions, induces tight junction damage and tight junction protein rearrangements. Chronic stress can shorten the duration to develop AD and dementia and increases its severity. APP, amyloid precursor protein; Aβ, beta amyloid; BBB, blood-brain barrier; GLUT-1, glucose transporter-1; IL, interleukin; NFTs, GVU, gliovascular unit; neurofibrillary tangles; NT, neurotensin; NVU, neurovascular unit; ZO, zonula occluden; ICAM-1, intercellular adhesion molecule-1; VCAM-1, vascular cell adhesion molecule-1.

Corticotropin-releasing hormone (CRH)/corticotrophin releasing factor (CRF) is expressed in neocortex, basal ganglia, amygdala and hippocampus in the CNS (Zhang et al., 2018). CRH released from the brain and peripheral system can activate mast cells to release neuroinflammatory mediators that can induce BBB permeability, neuroglial activation and neuroinflammation (Esposito et al., 2002; Theoharides and Konstantinidou, 2007; Figure 2). Mast cells express functional CRH-receptor1 (CRH-R1) and CRH-R2 receptors for CRH (Cao et al., 2005; Papadopoulou et al., 2005; Kritas et al., 2014). Mast cells can synthesize and release CRH that can activate mast cells and glial cells to release inflammatory mediators (Kempuraj et al., 2004; Yang et al., 2005). CRH-associated CRHR activation leads to neuronal death through protein kinase A (PKA), PKC, Ca^++^ and nuclear factor-kappa B (NF-kB) pathways in the neuroglia in the CNS disorders (Chen et al., 2014; Kritas et al., 2014). Stress-mediated CRH induces spine loss and inhibit synapse formation and inhibiting the dural secretion of chemokine (C-C motif) ligand 5 (CCL5) from glia (Zhang et al., 2018). CRH can directly affect brain endothelial cells and BBB permeability (Esposito et al., 2003). These reports indicate that stress can induce neuroinflammatory pathways.

## Stress Associated Changes in the BBB, Neurovascular Unit, Gliovascular Unit, Tight Junction, and Adherens Junction

About 600 km length of capillaries and micro vessels supply blood to the brain that consists of about 100 billion neurons. BBB is a special semi-permeable barrier that prevents, restricts and selectively allows the movement of cells and substances from the peripheral blood to the brain and brain to the blood (Stamatovic et al., 2016). BBB mainly maintains and protects healthy microenvironment in the brain, in addition to blood-cerebrospinal fluid barrier (BCSFB) and arachnoid barrier. Studies have shown the effects of acute and chronic stress on BBB dysfunctions, but the studies on specific molecular and ultrastructural changes at the tight junction proteins and adherens junction are insufficient, and thus not clearly understood (Santha et al., 2015). Oxygen, carbon dioxide, glucose, and amino acids can pass through BBB, but not any foreign objects, microorganism and toxins. BBB and tight junction proteins that exist between the vascular endothelial cells regulate the passage of large, negatively charged molecules through paracellular diffusion method, but the transcellular transportation across the endothelial cells is regulated by many transporter proteins, by endocytosis, and diffusion methods (Kealy et al., 2018).

Blood-brain barrier consists of non-fenestrated special type of endothelial cells, astrocytes, pericytes, innate immune cells, and basement membrane. BBB with neurons and astrocytes constitute NVU and GVU, respectively. Both neuroinflammation and stress conditions can affect NVU and GVU in the brain. The adjacent endothelial cells contact each other through tight junction that consists of occludin, claudin-1, claudin-3, claudin 5, claudin-12; zonula occludens (ZO) ZO-1, ZO-2, ZO-3; junctional adhesion molecule-A (JAM-A), JAM-B and JAM-C; adherens junction, and gap junction. Claudin-5 is the predominant type among claudins (Lochhead et al., 2017). BBB also includes adherens junction and gap junctions. Adherens junction consists of transmembrane proteins such as cadherens (Ve-cadherens, E-cadherens) and catenins (α catenin, β-catenin). Gap junction consists of many connexins such as connexin 37, connexin 40, and connexin 43. Additionally, endothelial cell-selective adhesion molecule (ESAM), partitioning defective-3 (Par-3), and Par-6 are other junction proteins similar to the JAMs (Stamatovic et al., 2016; Lochhead et al., 2017). Tight junction complexes such as claudins and occludins are connected intracellularly to the actin filaments. Both tight junction and adherens junction play different roles in BBB functions. Tight junction provides barrier functions and the adherens junction connects adjacent endothelial cells, promotes maturation of these cells, and provides plasticity to the endothelial cells. The junctional proteins can move and loose network connectivity in BBB dysfunctions in neurological and neuroinflammatory disorders. However, specific changes and relocation of junctional proteins in neurological disorders including AD and in stress is not yet clearly explored.

This emerging new field of research on BBB junctional complexes could provide useful information to understand the mechanism of neurological disorders such as in stress, stroke, dementia, and AD, and to develop disease specific and efficient therapeutic options. Pericytes are considered as the gatekeepers of the BBB and play role in angiogenesis and BBB integrity (Presta et al., 2018). Tight junctions prevent the flow of solutes through paracellular route. Substances such as glucose move across BBB through transcellular route by glucose transporter-1 (GLUT-1). GLUT-1 is highly expressed in the endothelial cells in the brain (DeStefano et al., 2018). Chronic social stress can cause BBB dysfunction associated with the loss of tight junction proteins such as claudin 5, and the entry of immune and inflammatory cells and cytokines from the peripheral system to the brain parenchyma (Menard et al., 2017).

Stress primarily affects hippocampus and frontal cortex in the brain. Study has shown that restraint stress significantly decreased claudin-5 and occludin in the hippocampus and frontal cortex in rats, at different periods of stress exposure (Santha et al., 2015). The same study also reported that restraint stress increased GLUT-1 and decreased astrocytic glial fibrillary acidic protein (GFAP) immunofluorescence in the frontal cortex. No neuronal changes were observed after immobilization stress, as determined by NeuN staining. Immobilization stress induces structural alterations of BBB endothelial cells. These endothelial cells show protrusions and detachment from the basement membrane (Santha et al., 2015). Immobilization stress increases the number of open junctions and damaged tight junctions, increases the thickness of the basal membrane, and edema of astrocytes in the hippocampus (Santha et al., 2015). Stress and aging contribute to the cognitive decline and hippocampal neurogenesis (Grilli, 2017).

Innate immune cells including granulocytes, macrophages, microglia and mast cells are important in the regulation of barrier functions of the BBB (Presta et al., 2018). Astrocytes, pericytes, and microglia release cytokines and chemokines that influence immune cells adhesion to the endothelial cells and migrate into the brain. However, the exact details of interactions and functions of these cells, and the ultrastructural and molecular mechanisms involved are not yet clearly understood. Acute stress can activate mast cells and increase the permeability of BBB (Kempuraj et al., 2017a). However, deficiency of mast cells or inhibition of mast cell activation by mast cell stabilizer disodium cromoglycate (Cromolyn) show reduced BBB permeability indicating mast cells play an important role in stress-induced BBB disruption (Esposito et al., 2001a,b). Activated mast cells release TNF-α that can downregulate the expression of the tight junction proteins such as occludin, claudin-5, ZO-1 and adherens junction VE-cadherin (Rochfort and Cummins, 2015). Other studies show that inhibition of TNF-α protects *in vitro* model of BBB that consists of endothelial cells and astrocytes, indicating the role of TNF-α in the BBB and tight junction dysfunctions (Abdullah et al., 2015; Rochfort and Cummins, 2015). A recent study show decreased expression of occludin and claudin 5 in the brain endothelial cells *in vitro* when incubated with mast cell tryptase (Zhou et al., 2018). Stress conditions alter BBB endothelial cells, tight junction proteins as well as the astrocytic end feet in neurodegenerative diseases including PD (Dodiya et al., 2018). Stress activates HPA axis through CRH and increases the release of glucocorticoids that inhibit immune response in the body (Esposito et al., 2001a). BBB dysfunction has been reported in many psychiatric disorders (Kealy et al., 2018). Mind and body practice such as yoga, exercise, nutritional supplement from plant products can reduce the level of pro-inflammatory mediators and improve the severity of pain, depression, anxiety, and cognition (Gu et al., 2018; Lurie, 2018). Stress is known to accelerate the onset and clinical severity of the experimental autoimmune encephalomyelitis (EAE) in mice in which mast cells are activated (Chandler et al., 2002; Brown and Hatfield, 2012). From the above reports, it is clear that stress affects BBB, NVU, and GVU in the brain.

## Stress and Alzheimer’s Disease

AD is an irreversible neurodegenerative disease characterized by the presence of extracellular APs, intracellular neurofibrillary tangles (NFTs) and hyperphosphorylated tau, neuronal loss, loss of synapses, NVU and GVU changes, and oxidative stress in the specific brain regions. About 5.7 million AD patients are currently living in the United States. AD is the sixth leading cause of death, and AD and AD dementia will cost $277 billion in the United States in 2018 (Alzheimer’s association, Chicago, IL, United States). Several chronic inflammatory conditions are associated with AD. There is no disease specific treatment option for AD, as the disease mechanism, risk factors, and the comorbid conditions are not yet clearly understood. Neuroinflammation, activation of glia, elevation of neuroinflammatory molecules and neuronal death are implicated in Alzheimer’s disease (Zaheer et al., 2008, 2011; Ahmed et al., 2017; Raikwar et al., 2018; Thangavel et al., 2018). Although the deposition of extracellular APs and intracellular formation of NFTs are traditionally considered as hallmarks of AD pathology over a long period, extensive recent findings indicate that several other factors including excessive neuroimmune and neuroinflammatory components significantly contribute to the pathogenesis of AD (Liberman et al., 2018; Saito and Saido, 2018). Therefore, the current drugs that target Aβ and NFTs did not show disease modifying beneficial effects, though they improve cognitive dysfunctions to some extent in AD patients (Fish et al., 2018). Newer approaches that target neuroimmune and neuroinflammatory components along with NVU and GVU are currently very active to treat neurodegenerative diseases including AD.

Chronic stress is one of the risk factors associated with dementia and AD pathogenesis (Rothman and Mattson, 2010; Bisht et al., 2018). It has been reported that mild and moderate stress conditions increase the level of amyloid precursor protein (APP), generation of Aβ peptide, intracellular NFTs, intracellular hyperphosphorylated tau, loss of synaptic plasticity, and extracellular APs that are associated with AD pathogenesis in the animals (Rothman and Mattson, 2010; Bisht et al., 2018; Justice, 2018; Figure 2). Chronic mild stress in APP_swe_/PS1_de9_ mice show depressive behaviors, reduced sociability, excessive Aβ level, glial cell activation and neuroinflammation in the brain (Gao et al., 2018). Another recent study showed that chronic noise stress altered gut microbiota, cognitive impairment, Aβ deposition in young senescence-accelerated mouse prone 8 (SAMP8) (Cui et al., 2018). Stress can exacerbate cognitive dysfunction and affect the functions of the hippocampus in the brain. Increased levels of Aβ is reported, even after 1 h of restraint stress. Further, stressful conditions such as modern life stress, chronic isolation stress, chronic social stress, chronic immobilization stress, and stress at early age show increased level of APs in the animals, indicating that these stressors are clearly associated with the pathogenesis of AD (Justice, 2018). Increased phosphorylation of tau and NFTs formation in many stress conditions lead to the damage of neurons and neuronal loss in AD and dementia (Sierra-Fonseca and Gosselink, 2018). The level of cortisol (corticosterone in rodents) is increased in stress conditions as well as in patients with dementia and cognitive impairment, indicating the relationship of stress and AD (Justice, 2018). One long term study, for over 50 years, with thirteen thousand patients reported that late-life depression increases the risk of dementia and AD (Barnes et al., 2012). Prolonged glucocorticoid levels in chronic stress can induce Aβ and tau deposition in AD pathogenesis in humans (Dong and Csernansky, 2009). Chronic stress also activates microglia that contribute to AD pathogenesis (Satoh et al., 2017). Gender and brain region-specific effects of stress has been reported previously (Devi et al., 2010; Bisht et al., 2018). Chronic restraint stress or repeated social defeat stress affects the release of neurotrophins and decreases the level of brain-derived neurotrophic factor (BDNF) that are important in neuronal growth, prevention of synaptic loss and maintenance of neuronal plasticity (Roth et al., 2011; Chiba et al., 2012). Pre-clinical stages in AD patients show reduced levels of pro-BDNF and BDNF (Peng et al., 2005). BBB dysfunction can activate β and γ secretase and generate and increase Aβ level in AD (Cai et al., 2018). Aβ transport through BBB is regulated by low density lipoprotein-1 (LRP-1) and receptor for advanced glycation end products (RAGE) expressed on the surface of the endothelial cells (Fei et al., 2018). Loss of pericytes and astrocyte abnormalities increases Aβ level in AD brains. Neurodegenerative diseases including AD show structural alterations in the tight junction proteins. Increased levels of RAGE associated Aβ toxicity induce damage to tight junctions in AD. BBB tight junction proteins ZO-1, occludin, claudin-1, claudin-3, claudin-5, claudin-12 and claudin-19 are implicated in AD pathogenesis. Occludin expression is increased in dementia and AD. Matrix metalloproteinases (MMPs) and apolipoproteinE4 (ApoE4) affect tight junction integrity in AD. Loss of tight junction integrity leads to increased permeability, edema, micro hemorrhage, and neuronal death (Yang et al., 2018). However, molecular and ultrastructural changes in BBB and tight junction proteins in AD is not yet clearly studied. Additionally, these changes in stress associated AD pathogenesis is much more complicated and currently not clearly understood. Moreover, studies also report that there is no association between Aβ and BBB dysfunctions. Aβ increases the expression of vascular adhesion molecules that are associated with the recruitment of inflammatory cells into the brain in AD. Increased hyperphosphorylation of tau generates NFTs that promote neuroinflammation, neuronal damage and BBB dysfunctions in AD. Childhood stress is associated with the development of dementia, cognitive impairment and neurodegeneration in late life in men (Donley et al., 2018). Further, a recent report indicate that early life stress is associated with late-onset-AD dementia (Lemche, 2018). However, it is not clear how the childhood stress continues to influence the body to develop neurological disorders in the late life. It is interesting to know if this effect is gender based or any population specific.

It has been shown that stress also increases cognitive dysfunctions in animals. Though several reports from animal studies support the concept that stress induces and worsens neuroinflammatory conditions including neurodegenerative diseases, the exact mechanism and the direct evidence are not yet clearly demonstrated, especially in the human diseases. This is because the exact mechanism of stress and AD pathogenesis is not yet clearly understood. Moreover, there are also significant differences in the stress response in the humans. Additionally, transgenic AD animal models show abnormal and aggressive behaviors with different degrees/severity of stress effects (Justice, 2018). Thus, animal models are not very suitable models to assess the stress effects that are much different in humans. It is very difficult to compare the results obtained from animal models of stress with the human patients due to these differences. Recently, it has been suggested that stress hormone CRH can be manipulated to reduce the risk of AD pathogenesis (Justice, 2018). Physical activities are associated with decreased risk of developing many chronic diseases in the aged. A recent study demonstrated that physical activity can reduce the chronic effects of restraint stress and the severity of AD in the animal model of AD (Yuede et al., 2018). There are several hypotheses and mechanisms proposed to explain how stress can accelerate AD pathogenesis. Aβ can activate neurons in the HPA axis that can induce stress effects and AD pathogenesis through cortisol. Mast cell activation-mediated inflammatory mediators play an important role in neuroinflammation (Kempuraj et al., 2016, 2017b; Hendriksen et al., 2017). Activation of neuroglia, inflammatory mediator release and neuroinflammation induce cognitive disorders, neurodegeneration and AD (Dansokho and Heneka, 2017; Swanson et al., 2018). We and others have previously reported that acute and chronic stress conditions can activate mast cells and that the increased mast cell activation can induce the onset and progression of neurodegenerative diseases including AD through the activation of neuroglia and increased BBB permeability (Shaik-Dasthagirisaheb and Conti, 2016; Kempuraj et al., 2017a). In fact, mast cell inhibitor drug Masitinib used as an adjunct therapy for mild to moderate AD in clinical trial has been shown to improve cognitive functions (Piette et al., 2011).

## Conclusion and Potential Future Development

Mast cells are associated with inflammation and pain. Stress conditions can activate mast cells and augment neuroinflammation through the activation of glial cells and neurons. Stress can induce HPA activation and mast cell activation that lead to neuroinflammation, BBB disruption and tight junction damage in the brain. Stress can induce the generation of APP, hyperphosphorylation of tau, NFTs, Aβ peptide, APs, oxidative stress, cognitive dysfunction, synaptic loss, neuronal loss, inflammatory mediator expression, and dementia in AD pathogenesis. Though several studies have shown the association of stress with BBB dysfunction, and tight junction protein alterations, the exact ultrastructural and molecular changes in these structures are not yet clearly known. Therefore, no effective therapeutic options are currently available to treat these conditions. Elaborate and sustained studies are needed to better understand these changes in stress associated AD pathogenesis in humans.

## Author Contributions

DK wrote and edited the manuscript. AZ critically edited the manuscript and acquired the funding. SM, RT, MA, GS, SPR, ID, SZ, and SI edited the manuscript.

## Conflict of Interest Statement

The authors declare that the research was conducted in the absence of any commercial or financial relationships that could be construed as a potential conflict of interest.
